# Improved Therapy of B-Cell Non-Hodgkin Lymphoma by Obinutuzumab-Dianthin Conjugates in Combination with the Endosomal Escape Enhancer SO1861

**DOI:** 10.3390/toxins14070478

**Published:** 2022-07-13

**Authors:** Hossein Panjideh, Nicole Niesler, Alexander Weng, Hendrik Fuchs

**Affiliations:** 1 Charité—Universitätsmedizin Berlin, Corporate Member of Freie Universität Berlin and Humboldt-Universität zu Berlin, Institute of Diagnostic Laboratory Medicine, Clinical Chemistry and Pathobiochemistry, Augustenburger Platz 1, D-13353 Berlin, Germany; hossein.panjideh@medicamentum.de (H.P.); n.niesler@xenikos.com (N.N.); 2 Institut für Pharmazie, Freie Universität Berlin, Königin-Luise-Straße 2+4, D-14195 Berlin, Germany; weng@zedat.fu-berlin.de

**Keywords:** targeted toxins, immunotoxins, obinutuzumab, anti-CD20, dianthin, glycosylated triterpenoids, endosomal escape, controlled drug release, cancer treatment, endocytosis

## Abstract

Immunotoxins do not only bind to cancer-specific receptors to mediate the elimination of tumor cells through the innate immune system, but also increase target cytotoxicity by the intrinsic toxin activity. The plant glycoside SO1861 was previously reported to enhance the endolysosomal escape of antibody-toxin conjugates in non-hematopoietic cells, thus increasing their cytotoxicity manifold. Here we tested this technology for the first time in a lymphoma in vivo model. First, the therapeutic CD20 antibody obinutuzumab was chemically conjugated to the ribosome-inactivating protein dianthin. The cytotoxicity of obinutuzumab-dianthin (ObiDi) was evaluated on human B-lymphocyte Burkitt’s lymphoma Raji cells and compared to human T-cell leukemia off-target Jurkat cells. When tested in combination with SO1861, the cytotoxicity for target cells was 131-fold greater than for off-target cells. In vivo imaging in a xenograft model of B-cell lymphoma in mice revealed that ObiDi/SO1861 efficiently prevents tumor growth (51.4% response rate) compared to the monotherapy with ObiDi (25.9%) and non-conjugated obinutuzumab (20.7%). The reduction of tumor volume and overall survival was also improved. Taken together, our results substantially contribute to the development of a combination therapy with SO1861 as a platform technology to enhance the efficacy of therapeutic antibody-toxin conjugates in lymphoma and leukemia.

## 1. Introduction

The cluster of differentiation-20 (CD20) is a surface protein mainly found on B-lymphocytes that is important for the differentiation and development of B-cells into plasma cells [1]. Increased expression of CD20 has been detected in patients with certain types of B-cell lymphoma and leukemia, making it an attractive target for antibody-based therapies [1]. Obinutuzumab (Gazyvaro^®^, Gazyva^®^) is a humanized and glycoengineered monoclonal IgG_1_ antibody against the CD20 type II epitope [2]. It is mainly applied for the treatment of chronic lymphatic leukemia in combination with chlorambucil, and follicular lymphoma in combination with bendamustine [3,4,5]. The type II mechanism of action together with glycoengineering of obinutuzumab results in augmented direct signaling-induced cell death as well as antibody-dependent cell-medicated cytotoxicity (ADCC) and phagocytosis (ADCP) while complement-dependent cytotoxicity (CDC) is diminished [4,6]. This distinguishes obinutuzumab from classical type I anti-CD20 monoclonal antibodies, such as rituximab and ofatumumab. Obinutuzumab initiates cell death through a non-apoptotic pathway that is dependent on actin rearrangement, lysosomal permeabilization, and reactive oxygen species generation. This pathway may potentially be exploited to eliminate malignant cells, which are refractory to conventional immunotherapy [7]. The immunogenic cell death is characterized by the release of damage-associated pattern molecules, such as heat shock protein 90 and adenosine triphosphate, and enhances the immune response by inducing dendritic cell maturation and subsequent T-cell activation [8].

Nevertheless, resistance to obinutuzumab-induced ADCC is a major problem for effective treatment. This is presumably caused by abnormal Fas signaling and can be overcome by combination therapies [9,10]. There are a large number of completed and ongoing studies of phase 1 to 3 with obinutuzumab to enable the application in further clinical indications and to increase efficacy by combining it with other anti-cancer drugs including acalabrutinib, bendamustine, chlorambucil, duvelisib, entospletinib, ibrutinib, idasanutlin, pixantrone, tirabrutinib, and venetocla [4,11,12,13]. For instance, 229 patients were included in a phase 3 study comparing obinutuzumab in combination with either chlorambucil or ibrutinib. At a median follow-up of 45 months, combination with ibrutinib significantly prolonged progression-free survival versus combination with chlorambucil (median not reached versus 22 months, *p* < 0.0001) [14]. 

An alternative to the combined application of an antibody with a toxic drug is the conjugation of both components to create antibody-drug conjugates (ADCs) or immunotoxins. There is no clear definition of these terms, and they are sometimes used as synonyms, but it has become commonly understood that ADCs are chemical conjugates of antibodies or derivatives thereof with cytotoxic small molecule drugs, while immunotoxins are antibodies that are chemically conjugated or genetically fused to protein toxins mainly obtained or derived from plant or bacterial origin [15,16]. The cytotoxic payload in ADCs can be linked via non-cleavable and cleavable linkers. Non-cleavable linkers have the advantage of being more resistant against degradation, and thus provide higher stability than cleavable linkers, while the latter are typically cleaved under particular environmental conditions of the cell, e.g., pH of endosomes, facilitating cytosolic entry [17]. In immunotoxins, the major function of the antibody is to specifically target the cancer cells, which does not exclude that the antibody also has effector functions, such as inducing apoptosis, CDC, and ADCC, as in the case of rituximab [18]. It has been reported that the ability of the antibody to bind to target cells and to interact with the innate immune system is not altered after chemical conjugation to toxins [19,20]. Therefore, the presence of an additional protein toxin can result in a higher efficacy than observed for sole antibodies that, in most cases, do not have the capacity for cell killing on their own, finally providing the potential for lower doses and less adverse events than observed for obinutuzumab [21]. 

An engineered immunotoxin (MT-3724) comprised of a modified cytotoxic Shiga-like toxin and a CD20-specific single-chain variable fragment [22] is currently tested in a clinical trial (NCT02361346). A more promising group of protein toxins is represented by type 1 ribosome-inactivating proteins (RIPs) such as saporin, dianthin, or gelonin that consist of a single catalytically active polypeptide chain (A chain), and are optimally suited for the design of targeted toxins since these toxins do not possess a natural cell-binding domain [23,24,25,26]. There are hundreds of RIPs and derived targeted toxins known [27,28].

The specific binding of immunotoxins to the receptors on target cells first triggers the mechanisms of action related to the antibody. In the case of obinutuzumab, ADCC is expected to be initiated after binding. Afterwards, immunotoxins are internalized by receptor-mediated endocytosis and enter into the endolysosomal trafficking pathway [29]. Here, the major problem comes to light: if the toxin remains entrapped in the endolysosomes, the toxin will be degraded, and the efficacy of the immunotoxins will be reduced [30]. Therefore, solutions are required that support the toxin moiety in escaping from the endolysosomes and entering the cytosol where it can cause cell death. Certain structurally specific plant glycosides are able to substantially enhance the endosomal escape of biological macromolecules [31]. One of them, SO1861, was shown to enhance the cytosolic uptake of both proteins and DNA, which can be present in soluble form, bound to magnetic nanoparticles, or polyplexed [32,33,34,35]. When the effector molecule reaches late endosomes and lysosomes, the low pH environment triggers the endosomal escape mediated by SO1861 [36]. The molecular mode of action is not known, but there are hints that the glucuronic acid functions as pH sensor. It is postulated that SO1861 mediates, once the glucuronic acid is protonated, a cholesterol depletion of the endosomal membranes, resulting in a loss of integrity [31,37].

To date, SO1861 has been investigated in vivo in mouse models only for targeted epidermal growth factor toxins to target solid non-metastatic tumors [32,38,39]. Immunotoxins have solely been examined in vitro in combination with SO1861. In a first study, immunotoxins that were created by chemically cross-linking the therapeutic antibodies cetuximab (anti-human epidermal growth factor 1) and trastuzumab (anti-human epidermal growth factor 2) to saporin caused specific enhanced cytotoxicity on tumor cells after co-application of SO1861 [20]. In a second study, the antibodies HB2 (anti-CD7), BU12 (anti-CD19), 4KB128 (anti-CD22), OKT10 (anti-CD38), and DF1513 (anti-CD71) were covalently attached to saporin, and the resulting immunotoxins exhibited augmented cytotoxicity in the presence of plant glycosides as well [40]. Rituximab had also already been coupled to saporin to create an immunotoxin. Some studies on Raji and D430B cells were conducted without endosomal escape enhancers [26,41,42] and another showed a 700-fold enhancement on Ramos cells in the presence of SO1861 [43]. To the best of our knowledge, immunotoxins of obinutuzumab or other anti-CD20 antibodies such as ofatumumab, tositumomab, and ibritumomab conjugated to RIPs are not yet described. Only an in-silico study of an immunotoxin composed of ofatumumab and the apoptosis inducing enzyme granzyme B is available [44].

In the present study, we tested for the first time the combination of an immunotoxin and SO1861 in a metastatic mouse cancer model. The stability and the rate of recombinant protein expression of dianthin encouraged us to use this ribosome-inactivating protein for conjugation with obinutuzumab. The objective of our study was to evaluate the therapeutic potential of obinutuzumab when applied as an immunotoxin in the presence and absence of an endosomal escape enhancer.

## 2. Results

### 2.1. Production of Obinutuzumab-Dianthin (ObiDi)

**Figure A1:**
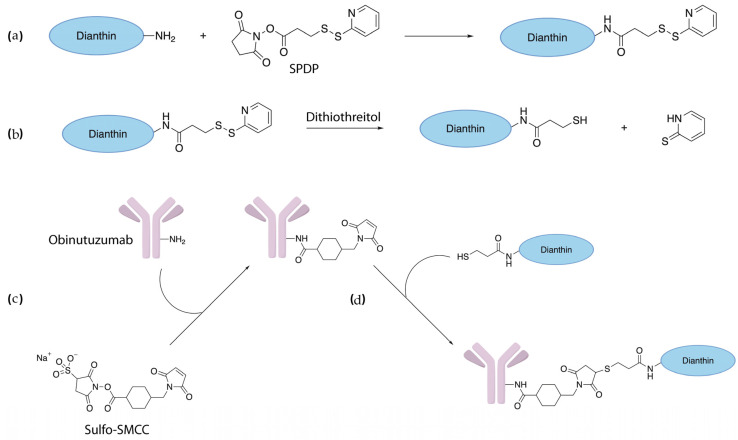
Chemical conjugation of obinutuzumab and dianthin. The toxin was attached to the antibody through a covalent bond introduced by the cleavable linker SPDP and non-cleavable Sulfo-SMCC in four reaction steps shown in panels (**a**) through (**d**).

Dianthin was covalently coupled to obinutuzumab via *N*-succinimidyl-3-(2-pyridyldithio) propionate (SPDP) and 4-(*N*-maleimidomethyl)cyclohexane-1-carboxylic acid 3-sulfo-*N*-hydroxysuccinimide ester (Sulfo-SMCC). The combination of these two cross-linkers introduces a non-cleavable covalent bond between the two proteins resulting in the formation of the immunotoxin ObiDi (Appendix A Figure A1). Although chemical cross-linking is an accepted and worldwide applied procedure, the major problem is the undirected reaction, which can result in large unusable aggregates. The balance between low conjugation yield and high aggregation was determined by the reaction conditions such as temperature, incubation time, and molar ratios of antibody, toxin, and cross-linker. 

**Figure 1:**
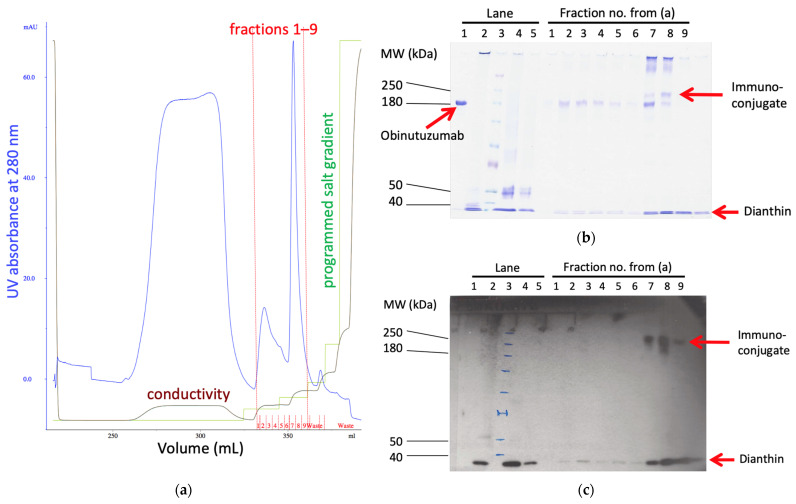
Purification of ObiDi. Step 1: Cation exchange chromatography to get rid of the parental antibody. (**a**) Elution profile (milli-absorbance at 280 nm versus elution volume, blue line) of the cation exchange chromatography with an optimized salt step gradient for elution. The two main peaks contained obinutuzumab (fractions no. 2–6) and ObiDi (fractions no. 7–9), red line. The green line represents the programmed gradient of NaCl (0–100% of buffer B) where buffer A is 20 mM HEPES buffer at pH 7.4 and buffer B is buffer A containing 2.0 M NaCl. The brown line represents the factual conductivity as a result of the salt gradient. (**b**) The fractions 1–9 obtained by cation exchange chromatography (**a**) were evaluated by (**b**) SDS-PAGE and (**c**) Western blotting with a His-tag-HRP antibody under non-reducing conditions. The different proteins are indicated by red arrows. Lane 1: obinutuzumab; lane 2: dianthin; lane 3: molecular mass marker; lane 4: obinutuzumab and dianthin after chemical conjugation but before cation exchange chromatography; lane 5: obinutuzumab and dianthin after chemical conjugation and after cation exchange chromatography (flow through). Unconjugated antibody does not contain a His-tag and can therefore not be visualized in the presented Western blot providing evidence that the high molecular mass bands represent conjugate. The conjugation reaction mixture (raw material) is too diluted to visualize the conjugate with Coomassie staining.

After first attempts of chemical conjugation, SDS-PAGE revealed that ObiDi appeared as defined conjugates with different drug-to-antibody ratios (DAR) (DAR 1–3 for the ratio of dianthin molecules per single obinutuzumab molecule), and in addition, as undefined aggregates at much higher molecular masses on the top of the gel. Unconjugated obinutuzumab was also visible while only minor amounts of dianthin were observed. After optimizing the reaction conditions in a large number of cycles, we were able to produce conjugates with a high content of DAR 1 and only minor amounts of aggregates. The next task was the removal of free obinutuzumab and free dianthin. In the first step, ObiDi was separated from unconjugated obinutuzumab and a part of unconjugated dianthin by cation exchange chromatography (Figure 1a). The elution profile showed a broad peak of non-bound material (flow throw) and mainly two distinct peaks. The smaller first peak represents unconjugated obinutuzumab whereas the larger sharp peak corresponds to the conjugate.

**Figure 2:**
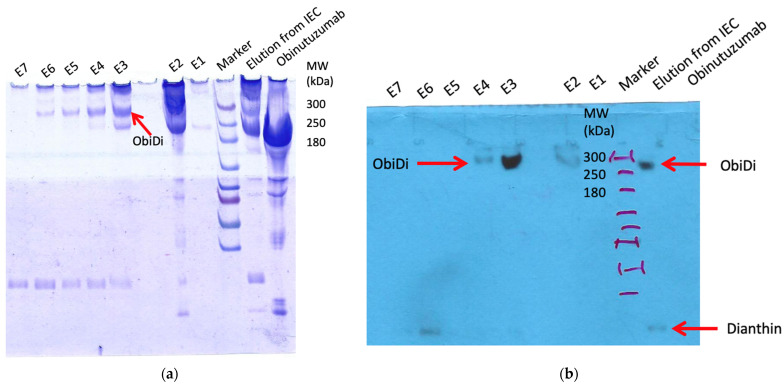
Purification of ObiDi. Step 2: Protein-A affinity chromatography to get rid of free dianthin. The conjugate obtained from cation exchange chromatography (lane “Elution from IEC”) was applied to the column and elution fractions E1 to E7 were collected. All samples were evaluated by (**a**) SDS-PAGE and (**b**) Western blotting with a His-tag-HRP antibody under non-reducing conditions. A conjugate of ObiDi with DAR 1 is visible in elution fractions E3 and E4. Free dianthin was successfully removed.

The presence of ObiDi in fractions 7−9 was confirmed by SDS-PAGE and the corresponding Western Blot (Figure 1b,c). The antibody used in the Western blot was directed against the His-tag of dianthin so that it was ensured that the protein detected at a high molecular mass of >200 kDa is the conjugate and not free antibody. However, free dianthin is still visible in fractions 7–9 in the Western blot, which can affect experiments with the conjugate in cell culture and in mice. Therefore, in a second step, ObiDi was separated from unconjugated dianthin by protein-A affinity chromatography. Here, unconjugated dianthin was directly eluted in the flow through whereas the purified immunoconjugate was clearly observed in fractions 3 and 4 after evaluation by SDS-PAGE and Western blotting (Figure 2). These fractions mainly contained ObiDi with DAR 1 and were then used for all further experiments on Raji and Jurkat cells and thereafter in the mouse lymphoma model. Yields were highly variable, as minute changes in reaction conditions resulted in significant losses due to aggregation (too much cross-linking) or insufficient conversion (too little cross-linking). Ultimately, the yield was between 1% and 10% related to the quantity of antibodies used. The enzymatic activity of conjugated dianthin was not determined here but is known to be reduced to 45–60% of the initial free dianthin [24,45,46,47], which is not crucial as cytotoxicity is maintained.

### 2.2. Cytotoxicity of ObiDi on Raji and Jurkat Cell Lines

**Figure 3:**
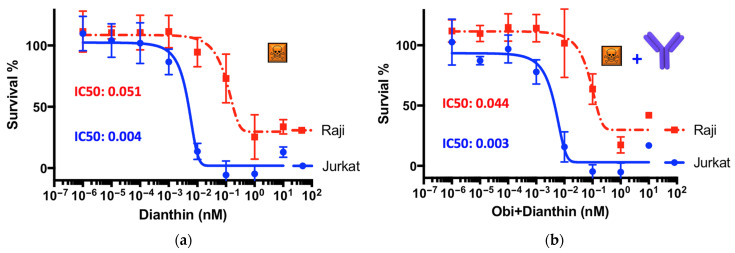
Undirected cytotoxicity of dianthin in the presence of the endosomal escape enhancer SO1861. Raji and Jurkat cells (20,000 cells/well) were seeded into a 96-well plate and allowed to grow for 24 h. They were then treated with (**a**) dianthin or (**b**) non-conjugated dianthin simply mixed with obinutuzumab. All proteins were applied at concentrations ranging from sub-femtomolar to upper nanomolar range. SO1861 was added at a final concentration of 1 µg/mL one hour before. Cells were incubated for 72 h in the presence of the compounds. Finally, cell proliferation was measured by an XTT assay and obtained values referred to untreated cells. Points in the graphs represent the mean ± SD of three biological experiments (*n* = 3) each one of them conducted in technical triplicate.

The cytotoxic effects of obinutuzumab, dianthin, and the conjugate ObiDi were evaluated in the presence of the endosomal escape enhancer SO1861 on Raji target cells and compared to off-target Jurkat cells. We started our experiments in the presence of SO1861 by treating the cells with free dianthin (Figure 3a) to investigate the undirected sensitivity of these different cell lines against this toxin. At the highest applied concentration of 100 nM, the cell viability of Raji cells was reduced to 25% compared to almost 0% for Jurkat cells. The concentration at which dianthin caused 50% growth inhibition (IC_50_) compared to the untreated control was calculated to 0.051 nM for Raji cells compared to 0.004 nM for Jurkat cells. This means that the off-target cells are about 10-fold more sensitive to ligand-free dianthin than the target cells. It is notable that this difference has nothing to do with the target receptor CD20 expression because dianthin does not carry a ligand here. The observed uptake of dianthin can potentially occur unspecifically via macropinocytosis and phagocytosis [48,49] by the binding of dianthin to low-density lipoprotein receptor-related proteins and subsequent clathrin-mediated endocytosis, as observed for the homologous protein saporin [50,51], or through receptor-independent endocytosis [52]. Once inside the cell, dianthin can be released from acidic compartments into the cytosol mediated by SO1861, which may explain the cytotoxicity of free dianthin. Due to the different genetic background and metabolism of Raji and Jurkat cells including type and rate of endocytosis, intracellular transport, and degradation potential, target receptor-independent uptake of dianthin can be substantially different from cell line to cell line. This has already been shown for a number of adherent cells [32] and is not of relevance for the tumor treatment for mainly two reasons. First, it is expected that the conjugates are relatively stable in serum so that free dianthin is cleaved off only in very small amounts, and second, the unspecific uptake is predominantly restricted to small proteins such as dianthin (30 kDa) and does not occur for large proteins as the conjugate (see below). For the conjugate, binding to the target receptor is much more important for successful uptake.

**Figure 4:**
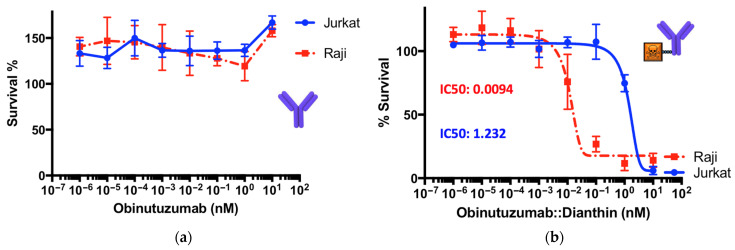
Cytotoxicity of (**a**) free obinutuzumab and (**b**) the conjugate ObiDi for target Raji and off-target Jurkat cells both in the presence of SO1861. The experiment was conducted as described in the legend of Figure 3. Each point in the graphs represents the mean ± SD of three biological experiments (*n* = 3) each one of them conducted in technical triplicate.

When obinutuzumab was applied together with non-conjugated dianthin, i.e., simply mixed, there was no significant difference observed in the IC_50_ values (Figure 3b). This means that there is no effective interaction between these two proteins and that free obinutuzumab does not contribute to an additional effect. This is confirmed by the mono-application of free obinutuzumab that does not show any cytotoxicity, either on Raji or on Jurkat cells (Figure 4a). The observation that obinutuzumab does not exhibit cytotoxic effects on target Raji cells is not surprising given the absence of human immune cells to mediate ADCC in this experiment. However, we have demonstrated in previous studies that antibodies as part of immunoconjugates, e.g., coupled to dianthin, retain their ability for ADCC when human immune cells are present [20]. Nevertheless, when the conjugate ObiDi was applied in the presence of SO1861, the IC_50_ for Raji cells was reduced to 0.0094 nM compared to an IC_50_ of 1.232 nM for Jurkat cells, pointing out that the cytotoxicity for target cells was 131-fold greater than for off-target cells. Two important conclusions can be drawn from these results. First, there is a clear therapeutic window for the treatment with ObiDi/SO1861 exhibiting an optimum at 100 pM where full toxicity is observed for target cells and no cytotoxicity for off-target cells. Second, the unspecific toxicity of dianthin for off-target cells is reduced due to the conjugation with the antibody.

### 2.3. Efficacy of ObiDi/SO1861 in a Lymphoma Mouse Tumor Model

**Figure 5:**
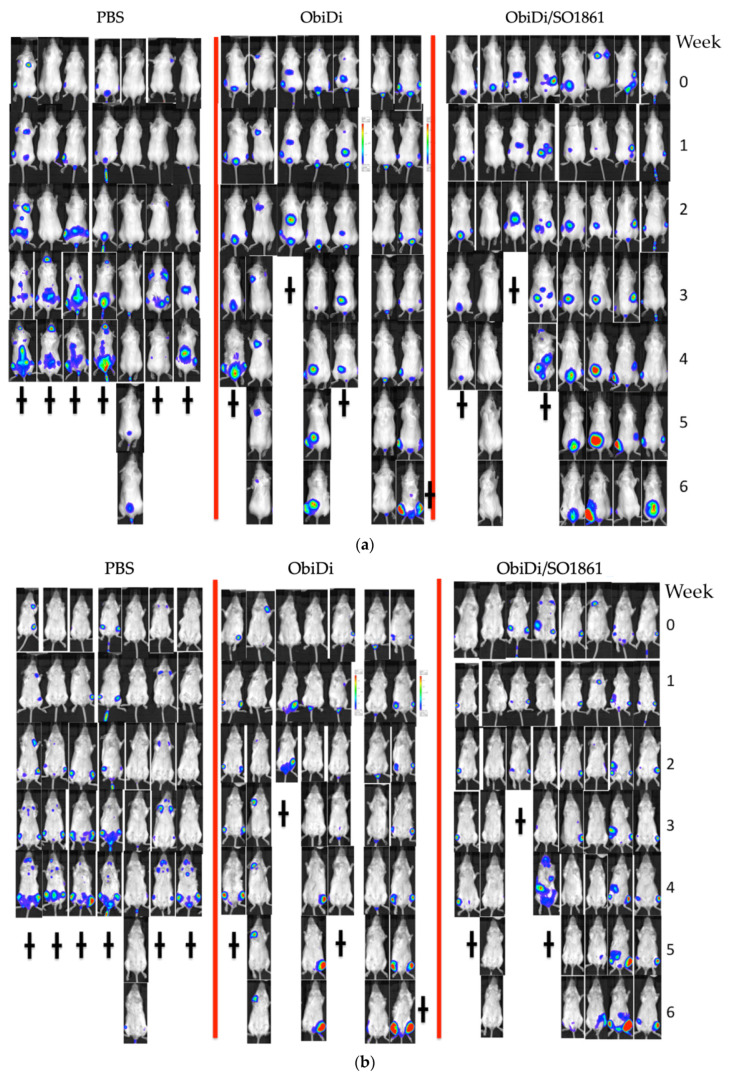

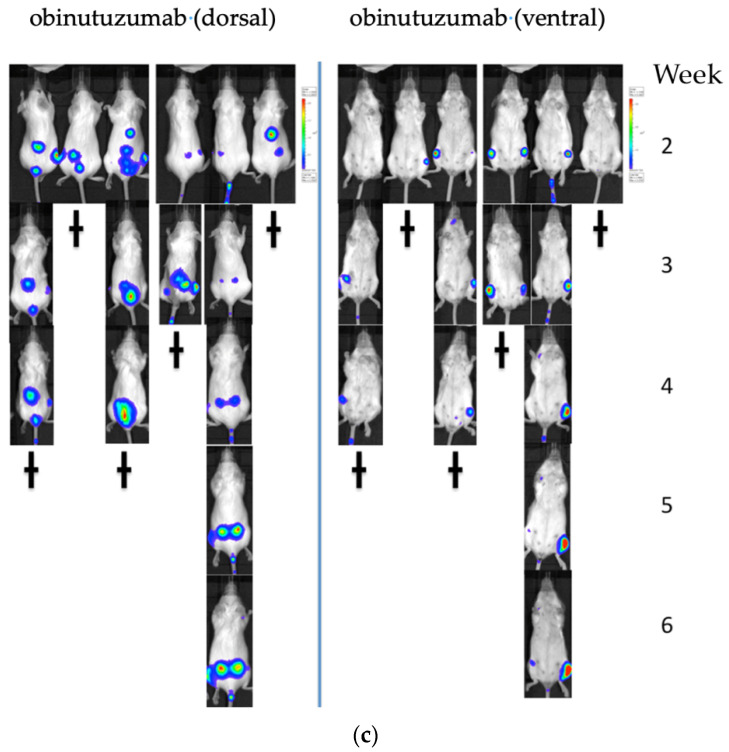
Examples for live imaging of metastases during treatment with either (**a**,**b**) ObiDi/SO1861, ObiDi, PBS or (**c**) obinutuzumab with (**a**,**c**) dorsal and (**b**,**c**) ventral view. The change in size of single metastases was quantified by using an IVIS Lumina imaging system 5 min after injecting luciferin intraperitoneally. The total flux values that correlate directly with tumor mass were measured weekly as indicated on the right side of the panels. The † indicates week of death.

**Figure A2:**
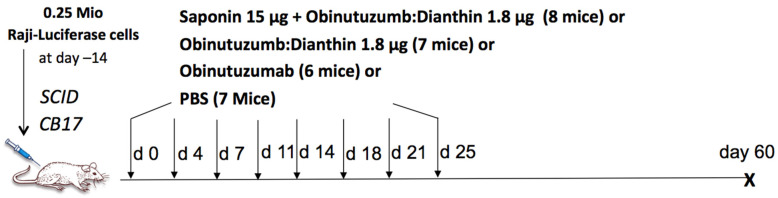
Experimental scheme of the treatment with ObiDi and obinutuzumab in a mouse lymphoma model. At day –14, SCID CB17 mice received luciferase-transduced Raji cells (0.25 × 10^6^) intravenously. Two weeks later, mice received either (i) 15 µg SO1861 in 100 µL PBS subcutaneously into the neck in combination with 1.8 µg ObiDi in 100 µL PBS intraperitoneally 1 h later, (ii) 1.8 µg ObiDi as in experiment (i), (iii) 1.8 µg Obinutuzumab in 100 µL PBS intraperitoneally or (iv) 100 µL PBS as mock-treated control. The mice were alternately treated every three and four days for four weeks. The live imaging to visualize tumors was conducted every week for six weeks.

The decisive question of the presented treatment method is, however, whether it also works in the living organism. Four mouse groups were included in the study, mock-treated control, monotherapy with toxin-free obinutuzumab as well as therapy with the conjugate ObiDi in the absence and presence of SO1861. The mice were inoculated with luciferase-producing Raji cells. This allowed us to visualize and track individual metastases over the whole period of the experiment. In a first experimental setting, we started treatment of the mice one day after tumor cell injection as described in many publications with similar objectives [53,54,55]. The aim of such type of experiment is to show that the appearance of detectable metastases is blocked or retarded, resulting in longer life span of the animals. When we applied this mouse model, already toxin-free obinutuzumab was very potent so that no tumors appeared in any of the animals of either group (except for the mock-treated control). Thus, this treatment regimen was not suitable to show superiority of ObiDi/SO1861 compared to obinutuzumab. The only conclusion that we obtained from this experiment is that neither the conjugation with dianthin nor the co-application with SO1861 obviously hampers the treatment with obinutuzumab. Therefore, we changed the procedure and did not start treatment before 14 days after tumor inoculation (Appendix A Figure A2). At that time, a large number of metastases of different sizes and locations were already observed in the animals (Figure 5).

We followed the growth and regression of individual metastases (ObiDi/SO1861, *n* = 35; ObiDi, *n* = 27; Obi, *n* = 29; PBS, *n* = 14) in all mice, and conducted a qualitative analysis (no remission, partial remission, complete remission) and quantitative analysis (regression to growth ratio). Metastases were identified as a single metastasis when they appeared as such in the image view because the image did not allow us to distinguish if it was a real single metastasis, two merged metastases, or two single metastases that overlapped in the projection. In all types of analyses, we clearly observed superiority of the ObiDi/SO1861 technique compared to the monotherapy with ObiDi. ObiDi in absence of SO1861 was only slightly better than the monotherapy with the parent antibody obinutuzumab. 

**Figure 6:**
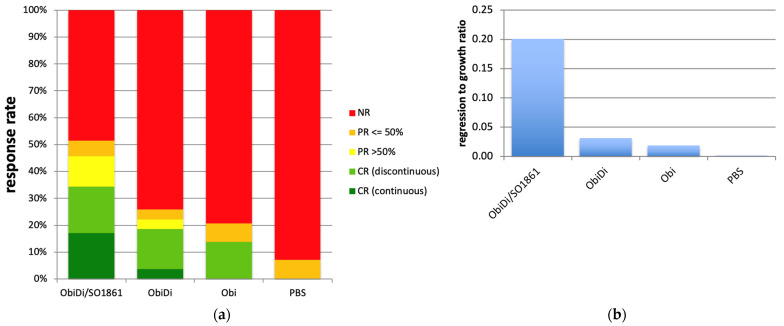
Treated mice indicating that the conjugate is well tolerated (Table 1). If abnormalities occurred in the mice, such as signs of paralysis, this always correlated with metastases affecting the central nervous system. There were no phenomena that could be directly assigned to the treatment.

The combination therapy of ObiDi with the endosomal escape enhancer SO1861 clearly prevents metastases growth better (51.4% response rate) than the monotherapy with ObiDi (25.9%) and obinutuzumab alone (20.7%) (Figure 6a). Continuous tumor regression was observed 4.6-fold more often in the combination therapy than in monotherapy with ObiDi and was not observed at all in sole obinutuzumab therapy. Partial regression of more than 50% was detected 3.1-fold more often in ObiDi/SO1861 than in ObiDi treatment, and was also not observed at all in obinutuzumab treatment. When looking at the total tumor quantity development expressed as the total regression to growth ratio during the whole observation period, ObiDi/SO1861 resulted in a 6.5-fold better tumor regression than ObiDi and a 10.6-fold better regression compared to obinutuzumab alone (Figure 6b). The ratio of 0.2 for the combination therapy nevertheless indicates that the total tumor growth was greater than tumor regression, however, it must be taken into consideration that the therapy started at a stage where the mice already had many metastases in the whole body, and a few of them developed quickly, thus substantially contributing to an increase in tumor mass. Six weeks after the beginning of the treatment, the overall survival rate was 71% for mice treated with ObiDi/SO1861, 57% for mice treated with ObiDi alone, 16% for those treated with obinutuzumab, and 14% for non-treated animals. Thus, in contrast to the immunotoxin, toxin-free obinutuzumab has no effect on survival when the treatment started not before 14 days after tumor cell inoculation, indicating that obinutuzumab is only highly effective when applied at an early stage of tumor cell proliferation. One mouse treated with ObiDi/SO1861 was completely cured and remained tumor free until the end of experiment. 

## 3. Discussion

The scientific community has been aware of immunotoxins for over 50 years. As early as 1970, Moolten and Cooperband described the selective destruction of target cells by a diphtheria toxin that was conjugated to antibodies directed against mumps antigens present on mumps-infected cells [56]. Six years later, Moolten et al. had already precisely defined the preconditions for effective immunotoxins and already recognized the importance of endosomal escape when they stated that intracellularly active toxins conjugated to antibodies must “undergo an internalization process that brings them, undegraded, to their intracellular sites of action” [57]. In the early 1980s, a variety of immunotoxins were developed [58] and some of them have also found their way into clinical studies [59]. Nevertheless, no ADCs or immunotoxins have been approved by the competent authorities in the last century (denileukin diftitox was approved in 1999, but was a cytokine conjugate and not an ADC), and only one ADC (gemtuzumab ozogamicin) was approved in the first decade of the current century. Progress in antibody design and production, and of site-specific conjugation techniques, led to the approval of 12 ADCs with small molecule drugs but only one immunotoxin (moxetumomab pasudotox) with a protein toxin as effector (truncated *Pseudomonas* exotoxin PE38) to date [60], indicating that endosomal escape is still a problem for the use of very efficient biological macromolecules.

At the beginning of this century, endosomal escape had come into the focus of controlled drug delivery techniques. The number of publications (PubMed) increased continuously from 27 in the period of 2002–2004 to 449 from 2019–2021. Various strategies have been investigated to make endosomes permeable for effector molecules. This includes the use of lysosomotropic agents, calcium channel antagonists, carboxylic ionophores, cell-penetrating peptides, and light-induced techniques [61]. A further option is the use of specific glycosides [31]. A glycoside with high endosomal escape enhancer potential is SO1861. The general effect was already observed in previous studies, mainly in growth-factor-targeted toxins [32,38,39], but also in immunotoxins applying the clinical antibodies rituximab, cetuximab, and trastuzumab, and other antibodies directed against CD22, CD25, and calcitonin receptor [20,25,33,43]. In contrast to the growth factor toxins, SO1861 was never tested in vivo together with immunotoxins, and in no case in a metastatic tumor model. 

The importance of the endosomal escape step was also shown for saporin, a RIP very similar to dianthin [62]. Geden et al. demonstrated that dimethylsulfoxide and lipopolyamines, which are known to disrupt the integrity of endosomal membranes, facilitated the rapid release of saporin from endosomes to the cytosol, while diphtheria toxin, ricin, or the catalytic A chain of ricin were not affected [63]. The same lack of the escape effect was also observed for SO1861 with ricin A chain and truncated versions of diphtheria toxin and *Pseudomonas* exotoxin A [46], indicating that the escape effect is restricted to specific cellular compartments and that intracellular trafficking of toxins essentially determines which toxins can benefit from endosomal escape enhancers.

We selected a disseminated cancer model with luciferase expressing Raji cells allowing quantitative measurement of single metastases. The target antigen CD20 is highly expressed on Raji cells while absent on Jurkat control cells [64]. Currently, there are four approved anti-tumor antibodies against CD20 on the market, obinutuzumab, ibritumomab, ofatumumab, and rituximab, and one further antibody, ocrelizumab, is approved to treat multiple sclerosis [60]. Obinutuzumab exhibits enhanced binding to low affinity immunoglobulin gamma Fc region receptor III and can induce in vitro an ADCC activity that is 35 to 100 times greater than that of ofatumumab and rituximab [65,66]. Clinical studies with obinutuzumab monotherapy showed up to 62% to 67% overall response rates but no significant differences in progression-free survival [67,68]. Therefore, obinutuzumab was combined with various other substances as described in the introduction. For instance, treatment with obinutuzumab in combination with chlorambucil increased progression-free survival to 27.7 months compared to the 16.3 months observed for rituximab/chlorambucil, and the rates of complete response were higher (20.7% versus 7.0%) [69]. Such success suggests that further emphasis should be placed on the use of obinutuzumab. On the other hand, treatments with obinutuzumab are also accompanied by adverse events including thrombocytopenia, infusion related reactions, cardiac events, and hepatotoxicity [21,60] and the efficacy also still has a large potential for improvement. ADCs and immunotoxins have the general potential to reach this goal with immunotoxins being even better when the problem of endolysosomal accumulation and degradation of the payload can be adequately addressed. 

In the present study, we therefore produced an immunotoxin consisting of obinutuzumab and the ribosome-inactivating protein dianthin and tested the conjugate in the presence and absence of the endosomal escape enhancer SO1861. Dianthin is a protein toxin with high efficacy inside cells but low toxicity in the circulation and is therefore well suitable for targeted tumor therapies [24]. The conjugate was compared with the obinutuzumab monotherapy. There are various factors that can affect the target and off-target cytotoxicity of the immunotoxin including the nature of the receptor, the specific epitope on the receptor, the level of receptor expression on target cells and off-target cells, the internalization process of the antibody-receptor complex, intracellular sorting/routing, the rate of endocytosis and recycling, the lysosomal activity, and endosomal escape [29,36,70,71,72]. Here, we cannot distinguish all these issues, but it is striking that ObiDi is around 300-fold less toxic for non-target Jurkat cells than free dianthin indicating that the small protein toxin dianthin can be unspecifically taken up by mechanisms such as pinocytosis while ObiDi cannot. Unspecific uptake of dianthin by Raji cells thus appears to be the explanation why the increase in cytotoxicity by ObiDi is only 5-fold compared to dianthin. Other issues such as a slow internalization rate of the CD20 target receptor might also play a role [73]. In vivo, it was observed that 30 µg of saporin, a RIP homologous to dianthin, is well tolerated by mice [74], indicating that free dianthin generated by cleavage of ObiDi is neglectable as the maximum amount of released dianthin is 0.3 µg if all conjugates have been split. The endosomal escape enhancer SO1861 itself is also non-toxic at the applied dose of 15 µg. Previous studies showed that SO1861 is well tolerated until doses of 100 µg, while 200 µg is lethal due to hemolytic effects [38].

Our results further demonstrate that the conjugation neither affects the binding activity of obinutuzumab nor the enzymatic activity in a sustainable manner. Moreover, our results clearly indicate that the combination of the conjugate ObiDi with SO1861 led to a synergistic improvement in the regression of CD20-sensitive metastases. As regards the slightly better efficacy of ObiDi compared to obinutuzumab in vivo, the experiments in the current study provide no hints as to which part of the toxic effect can be attributed to ADCC and which part to dianthin. Hypothetically, it could be the case that ADCC is completely lost in ObiDi treatment, and the toxic effect is fully caused by dianthin. However, it is most likely that both effects play a role. On the one hand, in a previous study, we already provided evidence that modified trastuzumab and cetuximab mediate efficient toxin delivery while ADCC is retained in target cells [20]. On the other hand, the drastic improvement of efficacy in the presence of SO1861 can only be explained by successful cytosolic delivery of catalytically active dianthin. Likewise known from previous studies [32,75,76], the enhancement by SO1861 was much higher on target cells than on off-target cells, resulting in a broadening of the therapeutic window. This allowed us to save treatment without observable drug-related adverse effects at low doses of 1.8 µg conjugate per application. All observed symptoms and early deaths were attributed to insensitive fast-growing metastases, mostly in the central nervous system. As early start of treatment resulted in complete suppression of tumor growth in all treatment groups (except mock-treated control), the potential of obinutuzumab alone appears to be high for tumors in early stages, while the superiority of the ObiDi/SO1861 therapy is particularly evident in advanced tumors.

## 4. Conclusions

In the present study, we focused on the efficacy of a therapeutic approach combining the endosomal escape enhancer function of the glycosylated triterpenoid SO1861 with the cytotoxic function of the antibody-targeted protein toxin obinutuzumab-dianthin to develop a new platform technology for the treatment of lymphoma and leukemia. Our results clearly demonstrated in vivo in a metastatic mouse tumor model that the application of ObiDi/SO1861 is substantially superior to the monotherapy with the conjugate ObiDi and the monotherapy with the parent antibody obinutuzumab. This study therefore suggests the usage of the ribosome-inactivating protein dianthin and SO1861 as a promising strategy to augment the efficacy of therapeutic antibodies in the treatment of lymphoma and leukemia, which should not be limited to obinutuzumab, thus opening further perspectives.

## 5. Materials and Methods

### 5.1. Recombinant Expression and Purification of Dianthin

The plasmid 6×His-tag-dianthin-pET11d [46] coding for dianthin was transformed into *Escherichia coli* Rosetta 2(DE3) pLysS Competent Cells (Novagen, San Diego, CA, USA). Bacteria were scaled up to a 2.0-L-culture with an optical density at 600 nm of 0.9 and expression of dianthin was induced by the addition of isopropyl β-D-1-thiogalactopyranoside (AppliChem, Darmstadt, Germany). Protein expression lasted for 3 h at 37 °C and 200 rpm. Bacteria were centrifuged at 5000× *g* and 4 °C for 5 min, resuspended in PBS and further purified by Ni-NTA chromatography (Protino Ni-NTA agarose, Macherey-Nagel, Düren, Germany), as described previously [77,78]. A bicinchoninic acid assay (Pierce/Thermo Scientific, Waltham, MA, USA) served to determine the protein concentration of the dianthin solution.

### 5.2. Chemical Conjugation and Purification of ObiDi

Purified dianthin was chemically conjugated to obinutuzumab (Gazyvaro^®^, Hoffmann-La Roche, Basel, Switzerland) through a covalent binding introduced by the linkers *N*-succinimidyl-3-(2-pyridyldithio) propionate (SPDP, Pierce/Thermo Scientific) and 4-(*N*-maleimidomethyl)cyclohexane-1-carboxylic acid 3-sulfo-*N*-hydroxysuccinimide ester (Sulfo-SMCC, Pierce/Thermo Scientific). First, 5.0 mg of obinutuzumab was dialyzed against phosphate-buffered saline (PBS) supplemented with 1 mM EDTA, 0.02% (*w*/*v*) sodium azide, pH 7.5. In parallel, 1.0 mg of dianthin was dialyzed against the same buffer. SPDP was added at a final concentration of 20 mM in Milli Q type 1 pure water to dianthin. Sulfo-SMCC was added at a final concentration of 14.7 mM to obinutuzumab solutions and again both solutions were dialyzed against the same buffer as before. Dithiothreitol (150 mM final concentration) was added only to the dianthin solution and reduction occurred for 30 min at 25 °C. Subsequently, dithiothreitol was removed from the dianthin solution by gel filtration (PD-10 desalting column, GE Healthcare, Uppsala, Sweden). Both obinutuzumab and dianthin solutions were mixed resulting in a molar ratio of 1:1 and the cross-linking reaction was allowed to occur for 18 h at 8 °C. 

ObiDi solution was concentrated to a volume of 5 mL and the buffer was changed to 20 mM HEPES pH 7.4 by an Amicon Ultra-15 (Merck Millipore, Carrigtwohill, Ireland) with 10,000 Da as nominal molecular mass limit (NMML). ObiDi was separated from unconjugated obinutuzumab and a part of unconjugated dianthin by cation exchange chromatography (SP High performance sepharose, GE Healthcare, Danderyd, Sweden). Bound proteins were eluted with a gradient of 1.5% to 10% of 2 M NaCl, 20 mM HEPES buffer with a constant flow rate of 0.5 mL/min at 18 °C. Fractions with protein content were analyzed by SDS-PAGE [7.5% (*w*/*v*) gel] under non-reducing conditions.

In a second step, the obtained ObiDi solution was separated from unconjugated dianthin by protein-A affinity chromatography according to the manufacturer’s instructions (Invitrogen). ObiDi purified via protein-A agarose was again concentrated by an Amicon Ultra-15 with 10,000 Da NMML and stored at 4 °C for its subsequent use. 

### 5.3. Cell Culture

Luciferase expressing Raji cells (human B-cell Burkitt’s lymphoma) were described previously [79]. Jurkat cells were purchased from the American Type Culture Collection (Manassas, VA, USA). Cells were cultured in RPMI-1640 medium with phenol red (PAA Laboratories, Pasching, Austria) supplemented with 20% fetal bovine serum (BioChrom KG, Berlin, Germany) and 1% penicillin/streptomycin (Gibco/Invitrogen, Karlsruhe, Germany). Cells were grown in humidified incubators at 5% CO_2_ and 37 °C.

### 5.4. Cytotoxicity Evaluation by XTT End-Point Assay

Cytotoxicity of ObiDi alone or in combination with SO1861 was evaluated on the CD20 positive Raji cell line and CD20 negative Jurkat cell line by the sodium 3′-[1-(phenylaminocarbonyl)-3,4-tetrazolium]-bis(4-methoxy6-nitro)benzene sulfonic acid hydrate (XTT, Serva Electrophoresis, Heidelberg, Germany) assay. Cells were seeded in a 96-well plate (20,000 cells/well) in 100 µL of RPMI-1640 medium without phenol red supplemented with 20% fetal bovine serum and 1% penicillin/streptomycin. After 24 h of cell proliferation, either 25 µL/well medium or 25 µL/well medium containing SO1861 at a final concentration of 1 µg/mL was added. Furthermore, 25 µL/well medium containing immunotoxin (ObiDi), obinutuzumab or dianthin was added at final concentrations from 0.1 fM to 100 nM. Raji and Jurkat cells were incubated in the presence of the compounds for further 72 h and finally cell proliferation was determined applying the XTT assay. A solution (50 µL/well) of XTT at 1 mg/mL and of phenazine-methosulfate (Serva Electrophoresis, Heidelberg, Germany) at 8 µg/mL was pipetted to the cells and the 96-well plate was incubated at 37 °C for 2 h. Absorbance was measured directly from the media at 450 nm by the SpectraMax 340PC Absorbance Microplate Reader (Molecular Devices, Sunnyvale, CA, USA). Untreated cells served as control and were set to 100% viability. IC_50_ values were calculated by four-parameter regression analysis using GraphPad Prism.

### 5.5. Raji B-Cell Lymphoma Xenotransplantation Model 

Eight-week-old female SCID CB17 mice (Charles River Laboratories, Germany) were inoculated intravenously with luciferase-expressing Raji lymphoma cells (0.25 × 10^6^) in 100 µL PBS. Paralysis of the hind legs or weight loss of >20% in the course of the experiments were used as end points. All animal experiments have been performed according to the institutional guidelines with permission from the competent authorities. The mice were housed in individually ventilated cages under a constant day and night cycle (12 h each) and had free access to water and animal feed. All animals were monitored daily for well-being during the entire experiment.

### 5.6. Immunotherapy of B-Cell Lymphoma Xenograft-Bearing Mice

Mice transplanted with luciferase-expressing Raji cells were randomly divided into groups of 6–8 animals. Two different settings were applied, (a) start of treatment one day after tumor cell inoculation as frequently described in the literature and (b) start of treatment two weeks later. Mice received either (i) 15 µg SO1861 in 100 µL PBS subcutaneously into the neck in combination with 1.8 µg ObiDi in 100 µL PBS intraperitoneally 1 h later, (ii) 1.8 µg ObiDi as in experiment (i), (iii) 1.8 µg obinutuzumab in 100 µL PBS intraperitoneally or (iv) 100 µL PBS as mock-treated control. The therapy lasted four weeks with alternating treatment intervals of 3 and 4 days.

### 5.7. Bioluminescent Imaging of Tumor Development 

Tumor development was quantified by in vivo imaging using an IVIS Lumina imaging system (Caliper-Life-Science, Waltham, MA, USA). Mice were anesthetized with isoflurane. Luciferin (Biosynth, Staad, Switzerland) was injected intraperitoneally with 375 mg/kg body weight and mice imaged 5 min later. Total flux values that correlate directly with tumor mass were measured weekly. Images were analyzed with Living Image 2.0 software (Caliper-Life-Science). 

### 5.8. Hematological Analysis

Four days after two treatments with 15 µg SO1861 in 100 µL PBS s.c. and 10 µg ObiDi in 100 µL PBS i.p. approximately 1.2 mL of blood was collected by cardiac puncture in isoflurane-anesthetized pretreated mice and compared with non-treated animals (*n* = 3). Blood was collected in S-Monovette (Sarstedt, Nümbrecht, Germany) 1.2-mL K3 EDTA sterile tubes and was analyzed by Labor 28 GmbH, Berlin, Germany, for blood parameters analysis, including cholinesterase, ferritin, glutamate dehydrogenase (GLDH), asparagine aminotransferase (ASAT), alanine aminotransferase (ALAT) and creatinine.

### 5.9. Isolation of SO1861

SO1861 was gained from the dried roots of the common soapwort *Saponaria officinalis* L. by methanol extraction and further purified by high-performance liquid chromatography, as described elsewhere [38,43,80].

## Figures and Tables

**Table 1:** Blood parameter of mice treated with ObiDi/SO1861 compared to untreated mice.

Parameter	Untreated	ObiDi/SO1861
alanine aminotransferase	46.7 ± 17.6 U/L	59.3 ± 22.5 U/L
asparagine aminotransferase	329.3 ± 180.4 U/L	248.3 ± 187.8 U/L
glutamate dehydrogenase	12.7 ± 5.1 U/L	16.3 ± 6.0 U/L
cholinesterase	5837.7 ± 223.4 U/L	6588.0 ± 548.4 U/L
creatinine	0.3 ± 0.0 mg/dL	0.2 ± 0.1 mg/dL
ferritin	n.d. ^1^	n.d. ^1^

^1^ n.d.: not detectable.

## Data Availability

All supporting data are provided in the Appendix A.
