# Correction: Panjideh et al. Improved Therapy of B-Cell Non-Hodgkin Lymphoma by Obinutuzumab-Dianthin Conjugates in Combination with the Endosomal Escape Enhancer SO1861. *Toxins* 2022, *14*, 478

**DOI:** 10.3390/toxins14100703

**Published:** 2022-10-13

**Authors:** Hossein Panjideh, Nicole Niesler, Alexander Weng, Hendrik Fuchs

**Affiliations:** 1 Charité—Universitätsmedizin Berlin, Corporate Member of Freie Universität Berlin and Humboldt-Universität zu Berlin, Institute of Diagnostic Laboratory Medicine, Clinical Chemistry and Pathobiochemistry, Augustenburger Platz 1, D-13353 Berlin, Germany; 2 Institut für Pharmazie, Freie Universität Berlin, Königin-Luise-Straße 2+4, D-14195 Berlin, Germany

The authors wish to make corrections to their paper [1].

In the original publication, there was a mistake in the legend for Figure 6 and Table 1 due to mistakes in the publication process. The original figure legend and part of the main text were missing, and Table 1 was placed before Figure 6, which was the wrong place. We wish to make the following corrections to this paper. The correct legend of Figure 6, main text between Figure 6 and Table 1, and Table 1 appear below.

**Figure 6:**
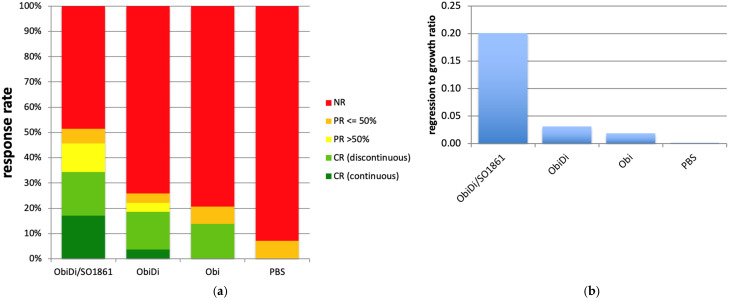
Analysis of metastases from live imaging. The tumor quantity of each metastasis was determined by bioluminescence using an IVIS Lumina imaging system. ObiDi/SO1861: combination therapy with obinutuzumab-dianthin conjugate and SO1861, *n* = 35; ObiDi: monotherapy with obinutuzumab-dianthin, *n* = 27; Obi: monotherapy with obinutuzumab, *n* = 29; PBS: mock-treated control, *n* = 14. (**a**) Response rates to metastases. NR (red): no remission, continuous tumor growth; PR ≤ 50% (orange): partial remission with tumor regression of maximal 50%; PR > 50% (yellow): partial remission with tumor regression of more than 50%; CR, discontinuous (light green): complete remission after initial or intermittent tumor growth; CR, continuous (dark green): complete remission with continuous tumor regression. (**b**) Ratio of regression to growth for the tumor quantity calculated as the sums of tumor mass loss and tumor mass gain over all metastases within each treatment group. If one of two equally sized metastases completely disappears during treatment and the other doubles in size, then the ratio for these two metastases is 1.0. The higher the ratio the better the success of the treatment.

Blood parameter analysis showed no significant differences between mock-treated and ObiDi/SO1861-treated mice indicating that the conjugate is well tolerated (Table 1). If abnormalities occurred in the mice, such as signs of paralysis, this always correlated with metastases affecting the central nervous system. There were no phenomena that could be directly assigned to the treatment.

**Table 1:** Blood parameter of mice treated with ObiDi/SO1861 compared to untreated mice.

Parameter	Untreated	ObiDi/SO1861
alanine aminotransferase	46.7 ± 17.6 U/L	59.3 ± 22.5 U/L
asparagine aminotransferase	329.3 ± 180.4 U/L	248.3 ± 187.8 U/L
glutamate dehydrogenase	12.7 ± 5.1 U/L	16.3 ± 6.0 U/L
cholinesterase	5837.7 ± 223.4 U/L	6588.0 ± 548.4 U/L
creatinine	0.3 ± 0.0 mg/dL	0.2 ± 0.1 mg/dL
ferritin	n.d. ^1^	n.d. ^1^

^1^ n.d.: not detectable.
